# Association of Hyperferritinemia With Distinct Host Response Aberrations in Patients With Community-Acquired Pneumonia

**DOI:** 10.1093/infdis/jiac013

**Published:** 2022-01-31

**Authors:** Xanthe Brands, Tjitske S R van Engelen, Floris M C de Vries, Bastiaan W Haak, Augustijn M Klarenbeek, Maadrika M N P Kanglie, Inge A H van den Berk, Alex R Schuurman, Hessel Peters-Sengers, Natasja A Otto, Daniël R Faber, René Lutter, Brendon P Scicluna, Jaap Stoker, Jan M Prins, W Joost Wiersinga, Tom van der Poll

**Affiliations:** Center for Experimental and Molecular Medicine, Amsterdam University Medical Centers, Location AMC, University of Amsterdam, Amsterdam, the Netherlands; Center for Experimental and Molecular Medicine, Amsterdam University Medical Centers, Location AMC, University of Amsterdam, Amsterdam, the Netherlands; Center for Experimental and Molecular Medicine, Amsterdam University Medical Centers, Location AMC, University of Amsterdam, Amsterdam, the Netherlands; Center for Experimental and Molecular Medicine, Amsterdam University Medical Centers, Location AMC, University of Amsterdam, Amsterdam, the Netherlands; Center for Experimental and Molecular Medicine, Amsterdam University Medical Centers, Location AMC, University of Amsterdam, Amsterdam, the Netherlands; Department of Radiology and Nuclear Medicine, Amsterdam University Medical Centers, Location AMC, University of Amsterdam, the Netherlands; Department of Radiology and Nuclear Medicine, Amsterdam University Medical Centers, Location AMC, University of Amsterdam, the Netherlands; Center for Experimental and Molecular Medicine, Amsterdam University Medical Centers, Location AMC, University of Amsterdam, Amsterdam, the Netherlands; Center for Experimental and Molecular Medicine, Amsterdam University Medical Centers, Location AMC, University of Amsterdam, Amsterdam, the Netherlands; Center for Experimental and Molecular Medicine, Amsterdam University Medical Centers, Location AMC, University of Amsterdam, Amsterdam, the Netherlands; Department of Internal Medicine, BovenIJ Hospital, Amsterdam, the Netherlands; Respiratory Medicine and Experimental Immunology, Amsterdam University Medical Centers, Location AMC, University of Amsterdam, Amsterdam, the Netherlands; Center for Experimental and Molecular Medicine, Amsterdam University Medical Centers, Location AMC, University of Amsterdam, Amsterdam, the Netherlands; Department of Clinical Epidemiology, Biostatistics and Bioinformatics, Amsterdam University Medical Centers, Location AMC, University of Amsterdam, Amsterdam, the Netherlands; Department of Radiology and Nuclear Medicine, Amsterdam University Medical Centers, Location AMC, University of Amsterdam, the Netherlands; Department of Internal Medicine, Division of Infectious Diseases, Amsterdam University Medical Centers, Location AMC, University of Amsterdam, Amsterdam, the Netherlands; Center for Experimental and Molecular Medicine, Amsterdam University Medical Centers, Location AMC, University of Amsterdam, Amsterdam, the Netherlands; Department of Internal Medicine, Division of Infectious Diseases, Amsterdam University Medical Centers, Location AMC, University of Amsterdam, Amsterdam, the Netherlands; Center for Experimental and Molecular Medicine, Amsterdam University Medical Centers, Location AMC, University of Amsterdam, Amsterdam, the Netherlands; Department of Internal Medicine, Division of Infectious Diseases, Amsterdam University Medical Centers, Location AMC, University of Amsterdam, Amsterdam, the Netherlands

**Keywords:** community-acquired pneumonia, sepsis, ferritin, biomarker, host response, immune suppression, systemic inflammation

## Abstract

**Background:**

Strongly elevated ferritin levels have been proposed to reflect systemic hyperinflammation in patients admitted to the intensive care unit. Knowledge of the incidence and pathophysiological implications of hyperferritinemia in patients with acute infection admitted to a non–intensive care setting is limited.

**Methods:**

We determined the association between hyperferritinemia, defined by 2 cutoff values (500 and 250 ng/mL), and aberrations in key host response mechanisms among patients with community-acquired pneumonia (CAP) on admission to a general hospital ward (clinicaltrials.gov NCT02928367; trialregister.nl NTR6163).

**Results:**

Plasma ferritin levels were higher in patients with CAP (n = 174; median [interquartile ranges], 259.5 [123.1–518.3] ng/mL) than in age- and sex-matched controls without infection (n = 50; 102.8 [53.5–185.7] ng/mL); *P* < .001); they were ≥500 ng/mL in 46 patients (26%) and ≥250 ng/mL in 90 (52%). Measurements of 26 biomarkers reflective of distinct pathophysiological domains showed that hyperferritinemia was associated with enhanced systemic inflammation, neutrophil activation, cytokine release, endothelial cell activation and dysfunction, and activation of the coagulation system. Results were robust across different cutoff values.

**Conclusions:**

Hyperferritinemia identifies patients with CAP with a broad deregulation of various host response mechanisms implicated in the pathogenesis of sepsis. This could inform future therapeutic strategies targeting subgroups within the CAP population.

## Teaser

Extremely elevated plasma ferritin levels have been linked to exaggerated systemic inflammation. Here we show that in patients hospitalized for community-acquired pneumonia, more moderate hyperferritinemia is associated with elevated levels of plasma biomarkers reflecting disturbances in key host response pathways.

Ferritin, a protein secreted mainly by hepatocytes, macrophages and Kupffer cells, is a well-known acute-phase reactant of which circulating levels increase in response to infection and other inflammatory conditions [1–4]. Hyperferritinemia has also been reported in more chronic disorders, such as cancer, liver diseases, metabolic syndrome, and kidney disease [5, 6]. Clinically, serum ferritin is used mainly as an indicator for iron status; low ferritin levels indicate iron deficiency elevated ferritin levels can be caused by multiple blood transfusions, hemochromatosis, or rare diseases such as hereditary hyperferritinemia, and very high levels are suggestive of Still disease and hemophagocytic lymphohistiocytosis [1, 7–9]. In patients with sepsis, ferritin has been proposed as a biomarker for macrophage activation–like syndrome (MALS), a hyperinflammatory condition associated with increased mortality rates [1, 10].

Community-acquired pneumonia (CAP) is the world’s leading infectious killer and the most common cause of sepsis [11, 12]. Our group previously reported that 83% of critically ill patients with sepsis caused by CAP have elevated plasma ferritin concentrations on admission to the intensive care unit (ICU) [4]. Ten percent of patients with CAP admitted to the ICU had ferritin concentrations >4420 ng/mL [4], the suggested cutoff for the diagnosis of MALS [1, 10]. In patients with CAP caused by severe acute respiratory syndrome coronavirus 2 (SARS-CoV-2), the cause of coronavirus disease 2019 (COVID-19), circulating ferritin has been used to classify hyperinflammation and is a predictor of a favorable response to treatment with recombinant interleukin 1 receptor antagonist (RA) [13]. Therefore, hyperferritinemia is used as an inclusion criterion to select patients with COVID-19 and exaggerated systemic inflammation; many trials use a ferritin level of ≥500 ng/mL to identify patients who might benefit from anti-inflammatory therapy (clinicaltrials.gov identifiers NCT04530578, NCT04341675, and NCT04443881).

Knowledge of the incidence of hyperferritinemia in (non-ICU) patients presenting to the hospital with CAP and its association with activation of distinct proinflammatory host response mechanisms is limited. In the current study, we sought to determine the association of hyperferritinemia in patients with CAP admitted to a general hospital ward with their clinical presentation, outcome, and aberrations in key host response pathways implicated in the immunopathology of pneumonia and sepsis.

## METHODS

### Study Population and Sample Collection

Patients were recruited as part of the ELDER-BIOME study (clinicaltrials.gov identifier NCT02928367) or OPTIMACT study (Dutch Trail Register identifier NTR6163), both approved by the medical ethical committees of the participating hospitals in the Netherlands: Amsterdam University Medical Centers, BovenIJ Hospital, and Spaarne Gasthuis Hospital [14–16]. Consecutive patients >18 years old admitted to 1 of the 3 hospitals between October 2016 and July 2018 were screened by trained (research) physicians. Patients were included if they were admitted with an acute infection of the respiratory tract, defined as ≥1 respiratory symptom (new cough or sputum production, dyspnea, chest pain, tachypnea, or abnormal lung examination findings) and 1 systemic symptom (fever [>38°C]) or hypothermia [<35.5°C], leukocytosis [leukocyte count >12 × 10^9^/L) or leukopenia (<4 × 10^9^/L), or elevated C-reactive protein [CRP] level [>30 mg/L]) and a new or progressive infiltrate, consolidation, cavitation, or pleural effusion seen with chest radiography or computed tomography [14, 16]. 

Patients were excluded if they had hospital-associated pneumonia or clinical suspicion of aspiration pneumonia [16]. Severity scores—the Pneumonia Severity Index (PSI) [17], the Modified Early Warning Score (MEWS) [18], and the quick Sequential Organ Failure Assessment (qSOFA) score [19]—were calculated on hospital admission. Age- and sex-matched subjects without acute infection served as controls. All participants, or their legal representatives, provided written informed consent and procedures followed were in accordance with the ethical standards of the Helsinki Declaration [20].

### Assays

Ethylenediaminetetraacetic acid (EDTA)–anticoagulated blood (for plasma biomarker measurements) was obtained within 16 hours of hospital admission. A subset of patients was also sampled 1 month after admission. We measured biomarkers reflective of key host response pathways using a Luminex multiplex assay (R&D Systems) and BioPlex 200 (BioRad). These biomarkers included ferritin, CRP, soluble triggering receptor expressed on myeloid cells (sTREM) 1, soluble CD163 (sCD163), and tenascin C (reflecting systemic inflammation); myeloperoxidase (MPO), proteinase 3, and neutrophil gelatinase-associated lipocalin (NGAL) (reflecting neutrophil activation); interleukin 6, 8, 10, 23, 27, and 1RA (cytokines); and soluble (s) E-selectin, soluble vascular cell adhesion molecule (sVCAM) 1, syndecan, endocan, sTie2, angiopoietin 1 and 2, von Willebrand factor, a disintegrin and metalloproteinase with a thrombospondin type 1 motif, member 13 (ADAMTS13), s-thrombomodulin, protein C, tissue factor pathway inhibitor (TFPI), and D-dimer (reflecting activation and function of the vascular endothelium and coagulation system). Leukocyte counts and differentials, as well as platelet counts, were determined in dipotassium EDTA–anticoagulated blood in the institutional hematology laboratory.

### Patient Stratification

In the primary analysis, patients were stratified using a ferritin level ≥500 ng/mL as the cutoff value, considering that several trials in patients with CAP caused by SARS-CoV2 use this value as an inclusion criterion (NCT04530578, NCT04341675, and NCT04443881). According to the literature, most hospital laboratories consider ferritin to be elevated when levels are >200 ng/mL in women and >300 ng/mL in men [6, 21]. Based on that, we stratified patients with CAP in our secondary analysis into those with normal (<250 ng/mL) or elevated (≥250 ng/mL) plasma ferritin levels.

### Statistical Analysis

Statistical analysis was performed within the R statistical framework (version 3.6.3; R Core Team 2020; R: A language and environment for statistical computing; R Foundation for Statistical Computing). All results are presented as numbers (percentages) for categorical variables, median and interquartile ranges (IQRs; Q1–Q3) for nonparametric quantitative variables, and means with standard deviations of the mean for parametric quantitative variables. Groups were compared as appropriate: continuous nonparametric data were analyzed using Wilcoxon signed rank sum or Kruskal-Wallis tests, categorical data using χ^2^ or Fisher exact tests, and continuous parametric data using Student *t* tests. Survival curves were compared using the log-rank test. Differences were considered statistically significant at *P* < .05 (Benjamini-Hochberg multiple-test corrected). 

## RESULTS

### Elevated Plasma Ferritin at Hospital Admission in Patients With CAP

**Figure 1.**
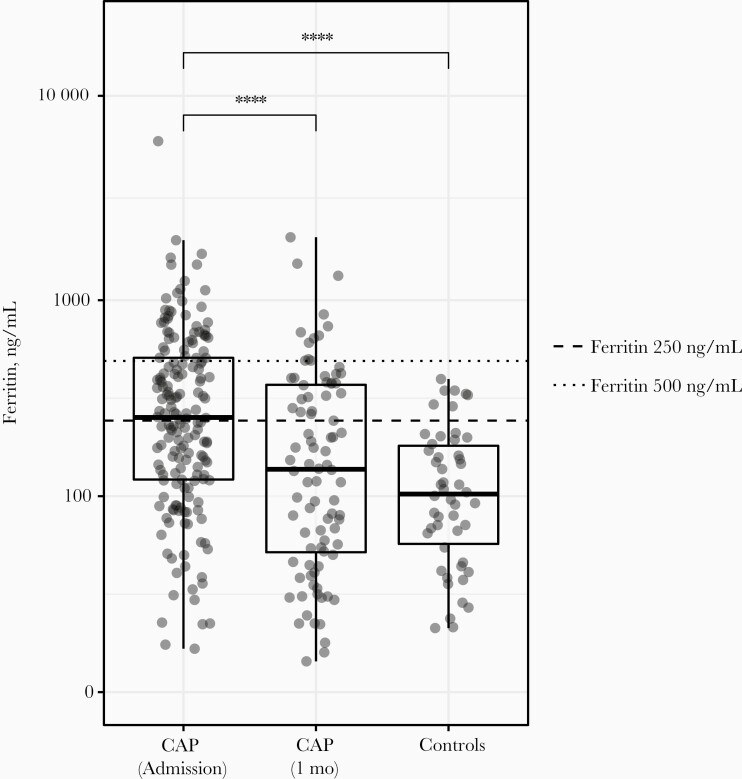
Patients with community-acquired pneumonia (CAP) have higher plasma ferritin levels than controls. Ferritin was measured in plasma samples from patients with CAP at admission (n = 174) and 1 month after admission (n = 88) and from noninfected age- and sex-matched controls (n = 50). Data are expressed as box-and-whisker plots, displaying individual data points and group medians (horizontal solid lines within boxes). Dotted line represents the ferritin cutoff of 500 ng/mL, used for primary analysis (ferritin ≥500 ng/mL, n = 46 patients); dashed line, cutoff of 250 ng/mL, used for secondary analysis (ferritin ≥250 ng/mL, n = 90 patients). Whiskers extend to the farthest point for values that are not outliers (ie, within 1.5 times the lower and upper bounds of the interquartile range). ∗∗∗∗*P < *.001 for difference between groups.

Between October 2016 and July 2018, 174 patients with CAP and 50 age- and sex-matched controls (mean age, 69 years; 58% male) without infection were enrolled. At hospital admission, patients with CAP had significantly higher plasma ferritin levels than noninfected controls (median [IQR], 259.5 [123.1–518.3] vs 102.8 [53.5–85.7] ng/mL, respectively; *P* < .001) (Figure 1). Plasma ferritin concentrations measured in a subset of patients with CAP (n = 88) approximately 1 month after admission (mean interval [standard deviation] 34 [6] days) (139.6 ng/mL; IQR 47.5–379.7) did not different significantly from those in controls (median [IQR], 139.6 [47.5–379] vs 102.8 [53.5–185.7] ng/mL, respectively; *P* = .12).

### Baseline Characteristics and Outcome in Patients With CAP Stratified by Plasma Ferritin Level at Admission 

Forty-six patients with CAP (26%) had a plasma ferritin concentration ≥500 ng/mL, compared with none of the controls (Figure 1). Among patients with CAP, those with plasma ferritin levels ≥500 ng/mL were comparable to those with levels <500 ng/mL, with regard to demographics and chronic comorbid conditions, although chronic obstructive pulmonary disease was less frequent and immunosuppression more frequent in the ≥500-ng/mL group (Table 1). Patients with plasma ferritin levels ≥500 ng/mL had higher PSI scores than those with levels <500 ng/mL, while the MEWS and qSOFA scores did not differ between groups. The rate of ICU admission and mortality rate at day 28 were low and did not differ between groups. A causative pathogen for CAP was detected in 67 of 128 patients (52%) with plasma ferritin levels <500 ng/mL and in 25 of 46 (54%) with levels ≥500 ng/mL (not different between groups; Supplementary Table 1). In both ferritin groups the most common pathogens were *Streptococcus pneumoniae, Haemophilus influenzae,* influenza virus A, and influenza virus B.

**Table 1.** Baseline Characteristics and Outcome in Patients With Community-Acquired Pneumonia Stratified According to Plasma Ferritin Concentration

Characteristic or Outcome	Low Ferritin (<500 ng/mL) (n = 128)	Elevated Ferritin (≥500 ng/mL) (n = 46)	*P* Value
Demographics			
Age, mean (SD), y	67.05 (17.39)	66.67 (13.20)	.90^a^
Female sex, no. (%)	68 (53.1)	27 (58.7)	.63^b^
BMI, median (IQR)^c^	25.32 (22.70–28.08)	25.70 (21.40–29.83)	.86^d^
Chronic comorbid condition, no. (%)			
COPD	41 (32.0)	7 (15.2)	.046^b^
Asthma	11 (8.6)	2 (4.3)	.54^b^
Cardiovascular disease^e^	40 (31.2)	9 (19.6)	.19^b^
Diabetes	39 (30.5)	11 (23.9)	.51^b^
Cancer	35 (27.3)	17 (37.0)	.30^b^
Hematological cancer	10 (7.8)	8 (17.4)	.12^b^
Solid tumor	26 (20.3)	10 (21.7)	>.99^b^
Neurological disease^f^	17 (13.3)	1 (2.2)	.07^b^
Chronic renal disease	14 (10.9)	6 (13.0)	.91^b^
Immune suppression^g^	26 (20.3)	18 (39.1)	.02^b^
Severity of disease at admission			
PSI score, median (IQR)	4.0 (3.0–60.0)	5.0 (4.0–77.0)	.04^d^
MEWS, median (IQR)	3.0 (2.0, 5.0)	3.5 (1.25–5.0)	.84^d^
qSOFA score, no (%)			
0	62 (50.8)	19 (44.2)	.71^b^
1	55 (44.3)	22 (48.8)	
2	6 (4.9)	3 (7.0)	
Blood cell counts, median (IQR), × 10^9^/L			
Leukocytes	11.90 (9.35–14.85)	10.80 (5.30–15.50)	.22^d^
Neutrophils	9.29 (7.13–12.19)	8.66 (4.99–15.71)	.69^d^
Lymphocytes	1.00 (0.64–1.43)	1.0 (0.55–1.83)	.63^d^
Monocytes	0.78 (0.60–1.18)	0.90 (0.36–1.23)	.72^d^
Thrombocytes	233.50 (173.25–287.00)	205.00 (155.50–335.50)	.54^d^
Neutrophil-lymphocyte ratio, median (IQR)	9.54 (5.88–14.30)	8.32 (4.74–18.88)	.67^d^
Outcome			
Length of hospital stay, median (IQR), d	4.0 (2.25–7.19)	5.75 (3.0–11.7)	.10^d^
ICU admission, no. (%)	8 (6.2)	7 (15.2)	.12^b^
Deaths within 28 d, no. (%)	6 (4.7)	3 (6.5)	.92^b^

Abbreviations: BMI, body mass index; COPD, chronic obstructive pulmonary disease; ICU, intensive care unit; IQR, interquartile range; MEWS, Modified Early Warning Score; PSI, Pneumonia Severity Index; qSOFA, quick Sequential Organ Failure Assessment; SD, standard deviation.

*P* value based on Student *t* test.

*P* values based on χ^2^ test.

BMI calculated as weight in kilograms divided by height in meters squared.

*P* values based Kruskal-Wallis test.

Cardiovascular disease included congestive heart failure, myocardial infarction, and peripheral vascular disease.

Neurological disease included cerebrovascular disease and dementia.

Including immunodeficiency (eg, human immune deficiency virus infection, AIDS, and asplenia) and use of immunosuppressive drugs (eg, corticosteroids, antineoplastic medication, and methotrexate).

### Enhanced Systemic Inflammatory, Cytokine, and Endothelial Procoagulant Responses in Patients With Ferritin Levels ≥500 ng/mL

**Figure 2.**
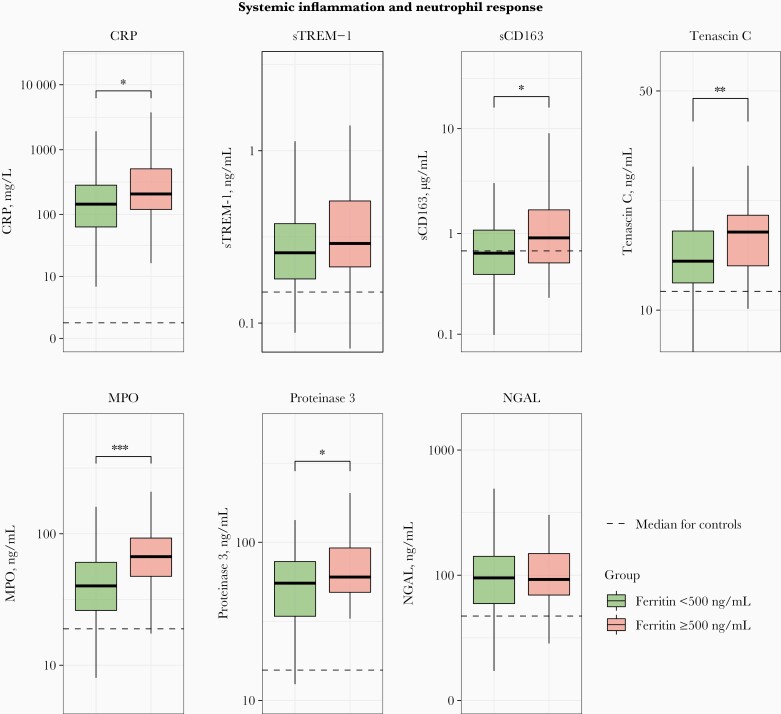
Systemic inflammatory and neutrophil responses in patients with community-acquired pneumonia, stratified according to plasma ferritin concentration (<500 ng/mL [n = 128] or ≥500 ng/mL [n = 46]). Data are expressed as box-and-whisker plots, with horizontal lines within boxes representing the group medians and tops and bottoms of boxes the upper and lower quartiles and whiskers extending to the farthest points that are not outliers (ie, within 1.5 times the lower and upper bounds of the interquartile range). Dotted lines represent median values obtained in 50 noninfected age- and sex-matched controls. ∗*P < *.05; ∗∗*P* < .01; ∗∗∗*P* < .001 (all Benjamini-Hochberg corrected). Abbreviations: CRP, C-reactive protein; MPO, myeloperoxidase; NGAL, neutrophil gelatinase-associated lipocalin; sCD163, soluble CD163; sTREM, soluble triggering receptor expressed on myeloid cells.

To obtain insight into the association between plasma ferritin concentrations ≥500 ng/mL and host response aberrations pertaining to pathophysiological pathways implicated in the pathogenesis of pneumonia and sepsis, we compared the plasma values for 26 protein biomarkers reflective of key host response domains between patients with ferritin levels ≥500 or <500 ng/mL. Of biomarkers indicative of systemic inflammation, CRP, sCD163, and tenascin C had higher levels in patients with admission ferritin levels ≥500 ng/mL, whereas sTREM-1 did not (Figure 2). Hyperferritinemia (ferritin ) was associated with higher plasma levels of the neutrophil degranulation products MPO and proteinase 3 (both constituents of azurophilic granules) but not higher levels of NGAL (derived from secondary granules) [22]; neutrophil counts did not differ between groups (Table 1). 

**Figure 3.**
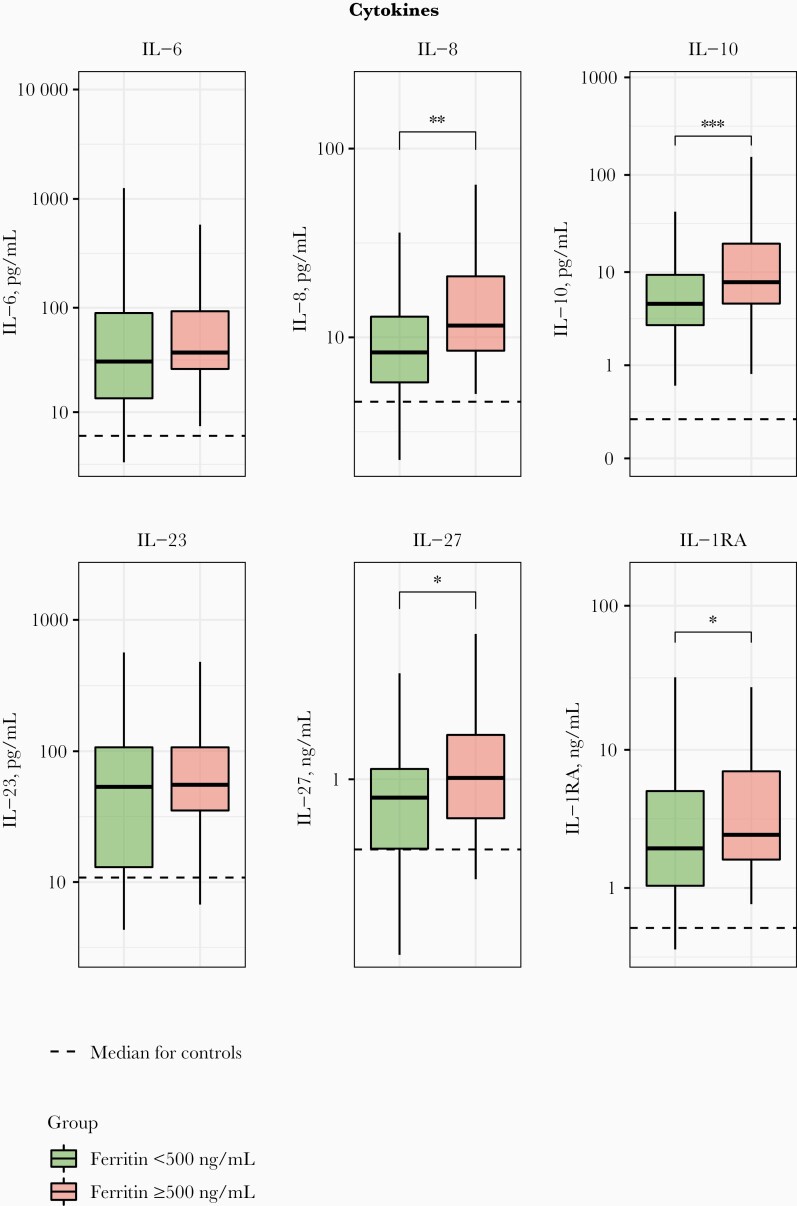
Plasma cytokine levels in patients with community-acquired pneumonia stratified according to plasma ferritin concentration (<500 ng/mL [n = 128] or ≥500 ng/mL [n = 46]). Data are expressed as box-and-whisker plots, with horizontal lines within boxes representing the group medians and tops and bottoms of boxes the upper and lower quartiles, and whiskers extending to the farthest points that are not outliers (ie, within 1.5 times the lower and upper bounds of the interquartile range). Dotted lines represent median values obtained in 50 noninfected age- and sex-matched controls. ∗*P < *.05; ∗∗*P* < .01; ∗∗∗*P* < .001 (all Benjamini-Hochberg corrected). Abbreviations: IL-6, IL-8, IL-10, IL-23, IL-27, and IL-1RA, interleukin 6, 8, 10, 23, 27, and 1 receptor antagonist, respectively.

**Figure 4.**
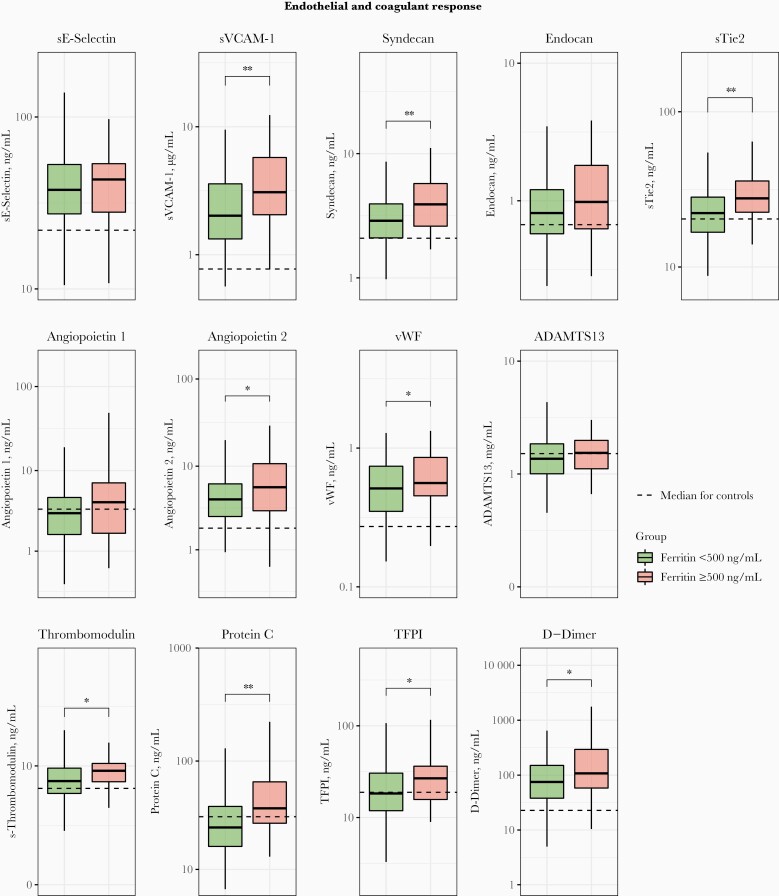
Endothelial and coagulant responses of patients with community-acquired pneumonia stratified according to plasma ferritin concentrations (<500 ng/mL (n = 128] or ≥500 ng/mL [n = 46]). Data are expressed as box-and-whisker plots, with horizontal lines within boxes representing the group medians and tops and bottoms of boxes the upper and lower quartiles, and whiskers extending to the farthest points that are not outliers (ie, within 1.5 times the lower and upper bounds of the interquartile range). Dotted lines represent median values obtained in 50 noninfected age- and sex-matched controls. ∗*P < *.05; ∗∗*P* < .01 (all Benjamini-Hochberg corrected). Abbreviations: ADAMTS13, a disintegrin and metalloproteinase with a thrombospondin type 1 motif, member 13; sE-selectin, soluble E-selectin; s-Thrombomodulin, soluble-Thrombomodulin; sTie2, soluble Tie2; sVCAM-1, soluble vascular cell adhesion molecule 1; vWF, von Willebrand factor; TFPI, tissue factor pathway inhibitor.

Patients with ferritin levels ≥500 ng/mL also showed exaggerated cytokine responses, as reflected by higher plasma levels of interleukin 8, 10, 27, and 1RA, while interleukin 6 and 23 levels did not differ between groups (Figure 3). In addition, patients with ferritin levels ≥500 ng/mL had higher plasma concentrations of biomarkers indicative of endothelial activation (sVCAM-1, von Willebrand factor, s-thrombomodulin, and TFPI), glycocalyx integrity (syndecan) and a more disturbed endothelial barrier function (sTie2 and angiopoietin 2); soluble E-selectin, endocan, ADAMTS13, and angiopoietin 1 did not differ between groups (Figure 4). Admission plasma ferritin levels ≥500 ng/mL were associated with enhanced activation of the coagulation system, as indicated by higher plasma levels of D-dimer; the anticoagulant proteins s-thrombomodulin, protein C, and TFPI also had higher levels in this high-ferritin group.

### Secondary Analysis With Hyperferritinemia Defined Using 250-ng/mL Cutoff 

In a secondary analysis, plasma ferritin levels ≥250 ng/mL (in most routine settings considered the upper limit of normal [6, 21]) were used as cutoff. Ninety patients with CAP (52%) and 7 noninfected controls (14%) had plasma ferritin levels ≥250 ng/mL (Figure 1). Patients with CAP with ferritin levels ≥250 or <250 ng/mL showed similar demographics. With regard to chronic comorbid conditions, hematological cancer, chronic renal disease and immune suppression were more frequent in those with levels ≥250 ng/mL, and chronic obstructive pulmonary disease less frequent (Supplementary Table 2). Severity scores (PSI, MEWS, and qSOFA) and clinical outcomes did not differ between patient groups. Differences in host response biomarker levels between patients with ferritin levels ≥250 or <250 ng/mL were largely similar to those in the primary analysis using a cutoff of 500 ng/mL (Supplementary Figures 1–3).

## DISCUSSION

Ferritin is an acute-phase protein elicited by inflammation and infection. Among acute conditions associated with hyperferritinemia, sepsis is the most common [3]. Knowledge of the frequency of hyperferritinemia in patients with acute infection is almost exclusively derived from ICU settings [2–4]. We here sought to assess the incidence of hyperferritinemia in patients with CAP hospitalized in a general ward and to determine its association with disturbances in key host response pathways implicated in the immunopathology of pneumonia and sepsis. We considered this of interest, as circulating ferritin levels have been and are used as inclusion criteria in trials evaluating immunomodulatory therapies in acute infections and may assist in identifying patients who might benefit from such therapies [13, 23] (clinicaltrials.gov identifiers NCT04530578, NCT04341675, NCT04443881, and NCT04990232). We performed 2 analyses, stratifying patients according to cutoff ferritin levels of ≥500 ng/mL (used in studies in patients with CAP caused by SARS-CoV2: NCT04530578, NCT04341675, and NCT04443881) or ≥250 ng/mL (around the normal reference value in most laboratories). While these cutoff levels are arbitrary, both analyses yielded highly similar results; that is, hyperferritinemia was associated with stronger aberrations in key host response pathways, including systemic inflammation, neutrophil activation, cytokine release, endothelial cell activation and dysfunction, and coagulation activation.

Several trials use a ferritin level of ≥500 ng/mL to select patients for anti-inflammatory therapy (clinicaltrials.gov identifiers NCT04530578, NCT04341675, and NCT04443881). Findings of the current study indicate that differences in host response biomarker levels using a ferritin cutoff level of 250 ng/mL were largely similar to those using a cutoff of 500 ng/mL. Notably, both cutoff levels are arbitrary, and strict recommendations regarding the optimal ferritin level for identification of patients who might benefit from anti-inflammatory therapy cannot be made based on our observational data.

Hyperferritinemia was associated with trends toward longer hospital stays and more frequent transfers to the ICU. Larger cohorts are needed to analyze whether hyperferritinemia in patients with CAP admitted to a general ward can be useful in predicting these clinically relevant outcomes.

In the current study there was no association between hyperferritinemia and the causative pathogen of CAP, neither for specific pathogens nor for bacterial and viral causes in general. Iron is an essential micronutrient for almost all living organisms, including bacteria, and several pathogens have developed mechanisms to extract iron from ferritin [24, 25]. Epidemiological studies have suggested an association between conditions with iron overload and an increased frequency of infections with a variety of microbes [26, 27], including Still disease [28] and hemochromatosis [29]. The vast majority of patients included in our study did not have comorbid conditions linked with hyperferritinemia. As such, the current data do not provide insight into the association between hyperferritinemia and increased susceptibility to pneumonia. Likewise, whether strongly elevated ferritin levels induced by infection facilitate microbial multiplication remains to be established.

Hyperferritinemia was modestly associated with severity of disease at hospital admission; only in the analysis using a cutoff of 500 ng/mL was the PSI score higher in patients with high ferritin concentrations, while the MEWS and qSOFA scores did not differ between groups. Nevertheless, most markers reflecting systemic inflammation had higher levels in patients with hyperferritinemia including sCD163 (like ferritin, considered a marker for macrophage activation) [1, 30]. MPO and proteinase 3 (neutrophil products derived from azurophilic granules) had higher plasma concentrations in patients with hyperferritinemia, while NGAL (a neutrophil protein derived from secondary granules) did not; notably, neutrophil counts did not differ between groups stratified according to ferritin levels, suggesting that hyperferritinemia may be a marker of neutrophil activation relating to azurophilic granule release. 

We measured a large set of endothelial cell biomarkers [31, 32] pointing at enhanced activation (higher sVCAM-1, von Willebrand factor, s-thrombomodulin and TFPI levels), more disturbed glycocalyx integrity (higher syndecan levels) and reduced endothelial barrier function (higher angiopoietin 2 levels) in patients with hyperferritinemia. Endothelial cell dysfunction has been suggested as a potential target in the treatment of sepsis [31, 32]; the current data suggest that hyperferritinemia may help identify patients who might benefit from vasculoprotective agents. D-dimer concentrations were higher in patients with hyperferritinemia, suggesting enhanced coagulation activation. The higher circulating levels of the endogenous anticoagulant proteins s-thrombomodulin and TFPI in the presence of hyperferritinemia likely reflect increased shedding from the activated vascular endothelium, which is expected to result in a loss of their cell-associated anticoagulant properties and to stimulate a procoagulant phenotype [31, 33].

The term *macrophage activation–like syndrome* (*MALS*) was introduced in recent years for a combination of clinical and laboratory features characterized by fever, hepatosplenomegaly, hepatobiliary dysfunction, coagulopathy, pancytopenia, and hypertriglyceridemia [1, 10]. Very high ferritin concentrations (>4420 ng/mL, ie, 8–17-fold higher than the cutoff values used in the present study) were proposed as a surrogate biomarker for MALS, with 97% specificity and 98% negative predictive value [1, 10]. The incidence of MALS in critically ill patients with sepsis was reported to vary between 3% and 4%, and the presence of MALS was independently associated with early death. 

Our group previously found a MALS incidence of 10%, defined by extreme hyperferritinemia (>4420 ng/mL), in patients with CAP admitted to the ICU [4]. MALS in critically ill patients with CAP was associated with increased mortality rate and exaggerated systemic inflammation, cytokine release, endothelial cell activation and coagulation activation [4], resembling the host response deviations reported here in non–critically ill patients with CAP stratified according to plasma ferritin levels. Notably, only 1 patient in the present study had ferritin levels >4420 ng/mL and the extent of host response dysregulation was much greater in patients with CAP in the ICU than in those not requiring intensive care.

While elevated circulating ferritin levels are used to select patients for immunomodulatory therapeutic strategies, literature on the pathophysiological implications of hyperferritinemia on the host response during acute infection is scarce. Findings of the current investigation, taken together with our group’s previous findings in critically ill patients [4], suggest that hyperferritinemia identifies patients with CAP with a broad deregulation of various key host response mechanisms implicated in the pathogenesis of sepsis, entailing not only systemic inflammation and cytokine release but also endothelial activation and dysfunction and activation of the coagulation system.

## Supplementary Data

Supplementary materials are available at *The Journal of Infectious Diseases* online. Supplementary materials consist of data provided by the author that are published to benefit the reader. The posted materials are not copyedited. The contents of all supplementary data are the sole responsibility of the authors. Questions or messages regarding errors should be addressed to the author.

jiac013_suppl_Supplementary_Table_S1

jiac013_suppl_Supplementary_Table_S2

jiac013_suppl_Supplementary_Figrue_S1

jiac013_suppl_Supplementary_Figrue_S2

jiac013_suppl_Supplementary_Figrue_S3

jiac013_suppl_Supplementary_Data

## Notes

***Acknowledgments.*** The authors thank all participants in this study, Rosan van der Lee for her aid in preprocessing the stimulation assays, Barbara S. Dierdorp and Tamara Dekker for their help with the cytokine and plasma biomarker measurements, and the OPTIMACT study group for providing the infrastructure for the OPTIMACT trial.

***Financial support.*** This work was supported by the Netherlands Organization for Health Research and Development (grants 505300098139 to X. B. and 40008129814016 to N. A. O. and Health Care Efficiency Program grant 843001806 for the OPTIMACT trial), the Amsterdam University Medical Centers (PhD scholarship to T. S. R. v. E.), Stichting de Merel (grant to T. S. R. v. E.), the Dutch Kidney Foundation (Kolff grant 19OK009 to H. P. S.), and the Amsterdam University Medical Centers (innovation grant).

***Potential conflicts of interest.*** All authors: No reported conflicts. All authors have submitted the ICMJE Form for Disclosure of Potential Conflicts of Interest. Conflicts that the editors consider relevant to the content of the manuscript have been disclosed.
