# Supplementary material for: Association of Hyperferritinemia With Distinct Host Response Aberrations in Patients With Community-Acquired Pneumonia
Source: J Infect Dis. 2022 Jan 31;225(11):2023–32. doi: 10.1093/infdis/jiac013 (PMC9312861; doi:10.1093/infdis/jiac013)
Supplement: jiac013_suppl_Supplementary_Table_S1 [file jiac013_suppl_supplementary_table_s1.docx]

**Supplementary Table 1: Causative pathogens of patients with CAP stratified according to a plasma ferritin concentration of <500 ng/ml or ≥500 ng/ml**

|  | **Low ferritin**  **(<500ng /ml)** | **Elevated ferritin**  **(≥500ng /ml)** | ***P* value** |
| --- | --- | --- | --- |
| Patients, *n* | 128 | 46 |  |
| Pathogen found | 67 (52.3) | 25 (54.3) | 0.951 |
| Bacteria | 36 (28.1) | 16 (34.8) | 0.510 |
| *Streptococcus pneumoniae* | 15 (11.7) | 7 (15.2) | 0.724 |
| *Heamophilus influenzae* | 9 (7.0) | 3 (6.5) | >0.99 |
| *Staphylococcus aureus* | 3 (2.3) | 1 (2.2) | >0.99 |
| *Pseudomonas aeruginosa* | 3 (2.3) | 0 (0.0) | 0.699 |
| *Pneumocystis carinii* | 0 (0.0) | 2 (4.3) | 0.117 |
| *Escherichia coli* | 1 (0.8) | 0 (0.0) | >0.99 |
| *Klebsiella pneumoniae* | 0 (0.0) | 1 (2.2) | 0.592 |
| *Legionella pneumophila* | 0 (0.0) | 1 (2.2) | 0.592 |
| *Mycobacterium tuberculosis* | 0 (0.0) | 1 (2.2) | 0.592 |
| Other bacterium^1^ | 7 (5.5) | 1 (2.2) | 0.614 |
| Viruses | 17 (13.3) | 12 (26.1) | 0.077 |
| Influenza A virus | 10 (7.8) | 2 (4.3) | 0.648 |
| Influenza B virus | 4 (3.1) | 3 (6.5) | 0.570 |
| Rhinovirus | 4 (3.1) | 0 (0.0) | 0.523 |
| Coronavirus (not SARS-Cov-2) | 3 (2.3) | 1 (2.2) | >0.99 |
| Respiratory syncytial virus | 2 (1.6) | 0 (0.0) | 0.963 |
| Parainfluenza 1-4 | 1 (0.8) | 1 (2.2) | >0.99 |
| Other virus^2^ | 1 (0.8) | 0 (0.0) | >0.99 |

^1^In ELDERBIOME: *Rothia dentocariosa* (*n* = 1), *Stenotrophomonas maltophilia* (*n* = 1), *Streptococcus pyogenes* (*n* = 1), *Mycoplasma pneumoniae* (*n* = 1); for OPTIMACT not further specified. ^2^Not present in ELDERBIOME; for OPTIMACT not further specified.
