# Supplementary material for: Association of Hyperferritinemia With Distinct Host Response Aberrations in Patients With Community-Acquired Pneumonia
Source: J Infect Dis. 2022 Jan 31;225(11):2023–32. doi: 10.1093/infdis/jiac013 (PMC9312861; doi:10.1093/infdis/jiac013)
Supplement: jiac013_suppl_Supplementary_Table_S2 [file jiac013_suppl_supplementary_table_s2.docx]

**Supplementary Table 2: Baseline characteristics and outcome of patients with CAP stratified according to a plasma ferritin concentration of <250 ng/ml or ≥250 ng/ml**

|  | **Normal ferritin**  **(<250ng /ml)** | **Elevated ferritin**  **(≥250ng /ml)** | ***p* value** |
| --- | --- | --- | --- |
| Patients, n | 84 | 90 |  |
| *Demographics* |  |  |  |
| Age, yr, mean (SD) | 67.07 (18.83) | 66.83 (13.75) | 0.924^‡^ |
| Sex, female, n (%) | 40 (47.6) | 55 (61.1) | 0.102^†^ |
| Body Mass Index, median [IQR] | 25.54 [22.70, 29.81] | 25.12 [22.08, 27.91] | 0.42* |
| *Chronic comorbidity, n (%)* |  |  |  |
| COPD | 30 (35.7) | 18 (20.0) | 0.032^†^ |
| Asthma | 7 (8.3) | 6 (6.7) | 0.897^†^ |
| Cardiovascular disease^1^ | 29 (34.5) | 20 (22.2) | 0.102^†^ |
| Diabetes | 24 (28.6) | 26 (28.9) | >0.99^†^ |
| Malignancy | 23 (27.4) | 29 (32.2) | 0.595^†^ |
| Hematologic malignancy | 4 (4.8) | 14 (15.6) | 0.037^†^ |
| Solid tumor | 19 (22.6) | 17 (18.9) | 0.675^†^ |
| Neurological disease^2^ | 12 (14.3) | 6 (6.7) | 0.162^†^ |
| Chronic renal disease | 5 (6.0) | 15 (16.7) | 0.048^†^ |
| Immune suppression^3^ | 13 (15.5) | 31 (34.4) | 0.007^†^ |
| *Severity of disease on admission* |  |  |  |
| PSI, score, median [IQR] | 4.0 [3.0, 65.25] | 4.0 [4.0, 68.0] | 0.366* |
| MEWS, median [IQR] | 3.0 [2.0, 5.0] | 3.5 [1.25, 5.0] | 0.842* |
| qSOFA, score, n (%) |  |  | 0.778^†^ |
| 0 points | 42 (51.9) | 39 (46.4) |  |
| 1 point | 35 (43.2) | 40 (47.6) |  |
| 2 points | 4 (4.9) | 5 (6.0) |  |
| *Hematology* |  |  |  |
| Leukocytes ×109, median [IQR] | 11.80 [9.05, 14.07] | 11.85 [7.80, 17.08] | 0.652* |
| Neutrophils ×10^9^, median [IQR] | 9.18 [7.04, 11.53] | 9.20 [6.05, 15.14] | 0.66* |
| Lymphocytes ×10^9^, median [IQR] | 1.00 [0.69, 1.42] | 0.95 [0.57, 1.70] | 0.942* |
| Monocytes ×10^9^, median [IQR] | 0.80 [0.60, 1.19] | 0.77 [0.51, 1.20] | 0.519* |
| Ratio neutrophils : lymphocytes | 10.43 [5.69, 17.50] | 9.08 [5.59, 14.11] | 0.529* |
| Thrombocytes ×10^9^, median [IQR] | 249.00 [185.25, 295.00] | 201.00 [156.00, 273.50] | 0.022* |
| *Outcome* |  |  |  |
| Length of hospital stay, days, median [IQR] | 4.0 [2.25, 6.81] | 5.25 [3.0, 9.75] | 0.051* |
| ICU admission, n (%) | 6 (7.1) | 9 (10.0) | 0.689^†^ |
| Mortality 28 days, n (%) | 5 (6.0) | 4 (4.4) | 0.925^†^ |

^1^ Congestive heart failure, myocardial infarction, peripheral vascular disease; ^2^ Cerebrovascular disease, dementia; ^3^ Immune deficiency (such as human immune deficiency virus (HIV) infection, acquired immune deficiency syndrome (AIDS), asplenia), use of immune suppressive drugs (such as corticosteroids, antineoplastic medication, methotrexate). COPD: Chronic Obstructive Pulmonary Disease; PSI: Pneumonia Severity Index; MEWS: Modified Early Warning Score; qSOFA: quick Sequential Organ Failure Assessment score; bpm: breaths/beats per minute; mmHg: millimeter of mercury. * Kruskal-Wallis test probability † Chi-square test probability ‡ Student t-test
