# Supplementary figures and images for: Association of Hyperferritinemia With Distinct Host Response Aberrations in Patients With Community-Acquired Pneumonia
Source: J Infect Dis. 2022 Jan 31;225(11):2023–32. doi: 10.1093/infdis/jiac013 (PMC9312861; doi:10.1093/infdis/jiac013)

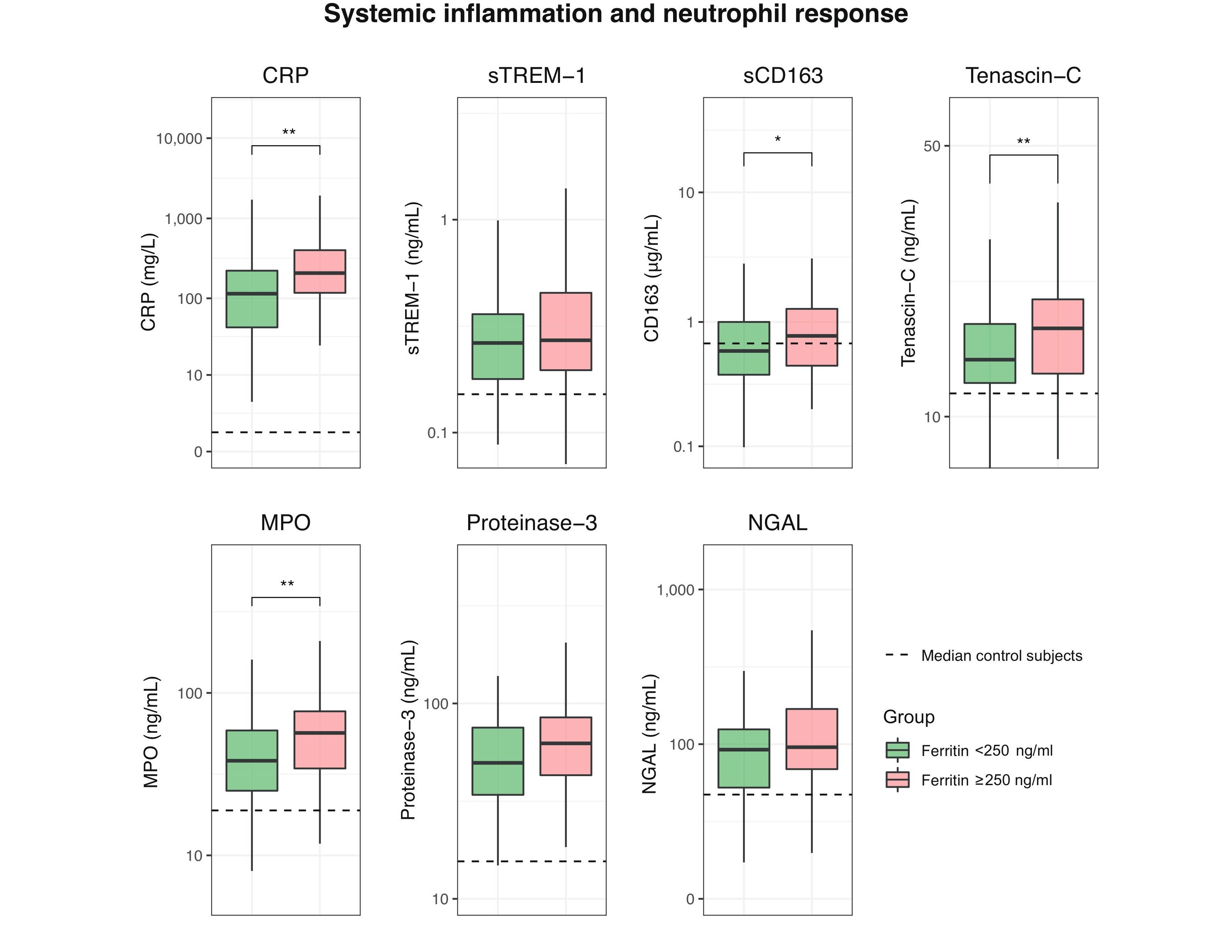

Supplement: jiac013_suppl_Supplementary_Figrue_S1 [file jiac013_suppl_supplementary_figrue_s1.jpeg]

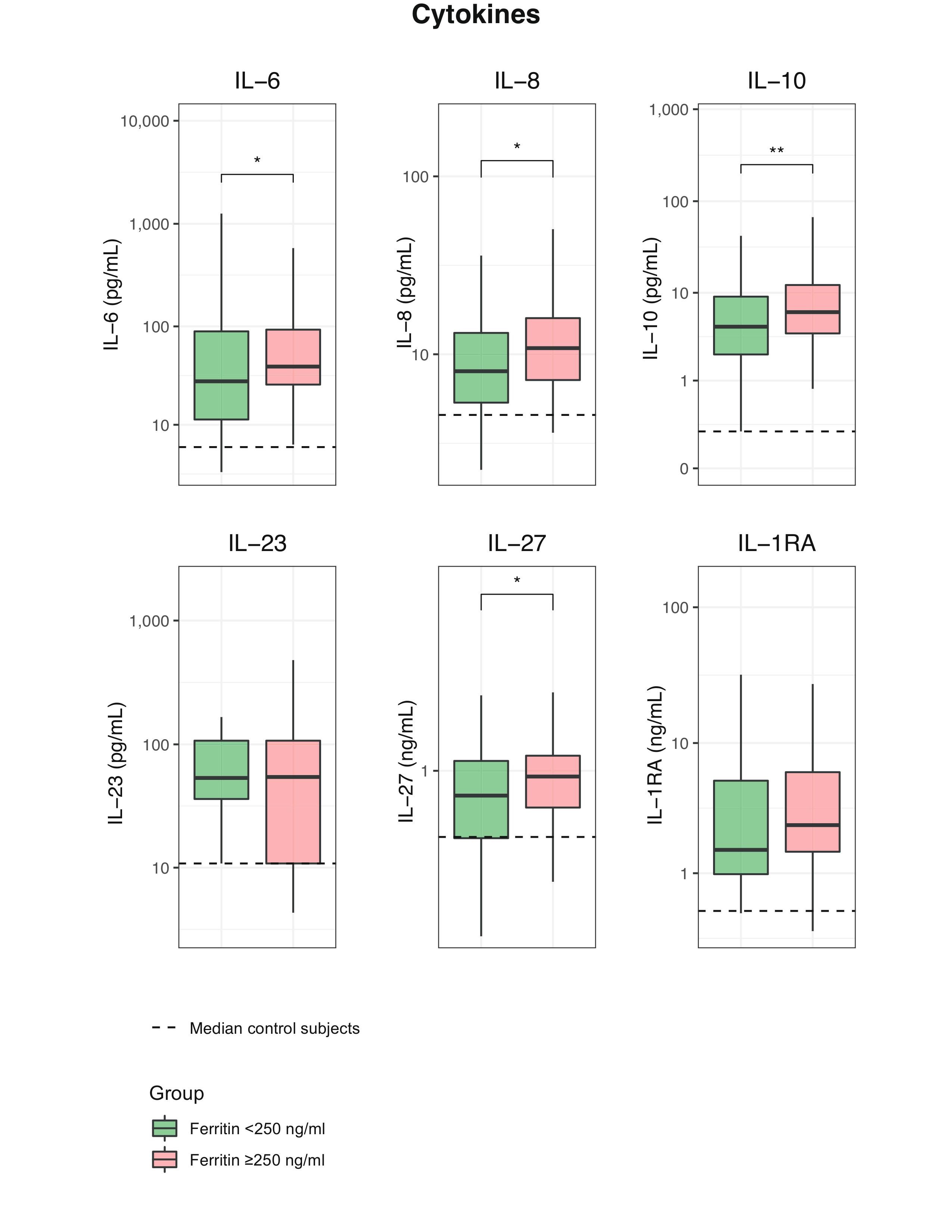

Supplement: jiac013_suppl_Supplementary_Figrue_S2 [file jiac013_suppl_supplementary_figrue_s2.jpeg]

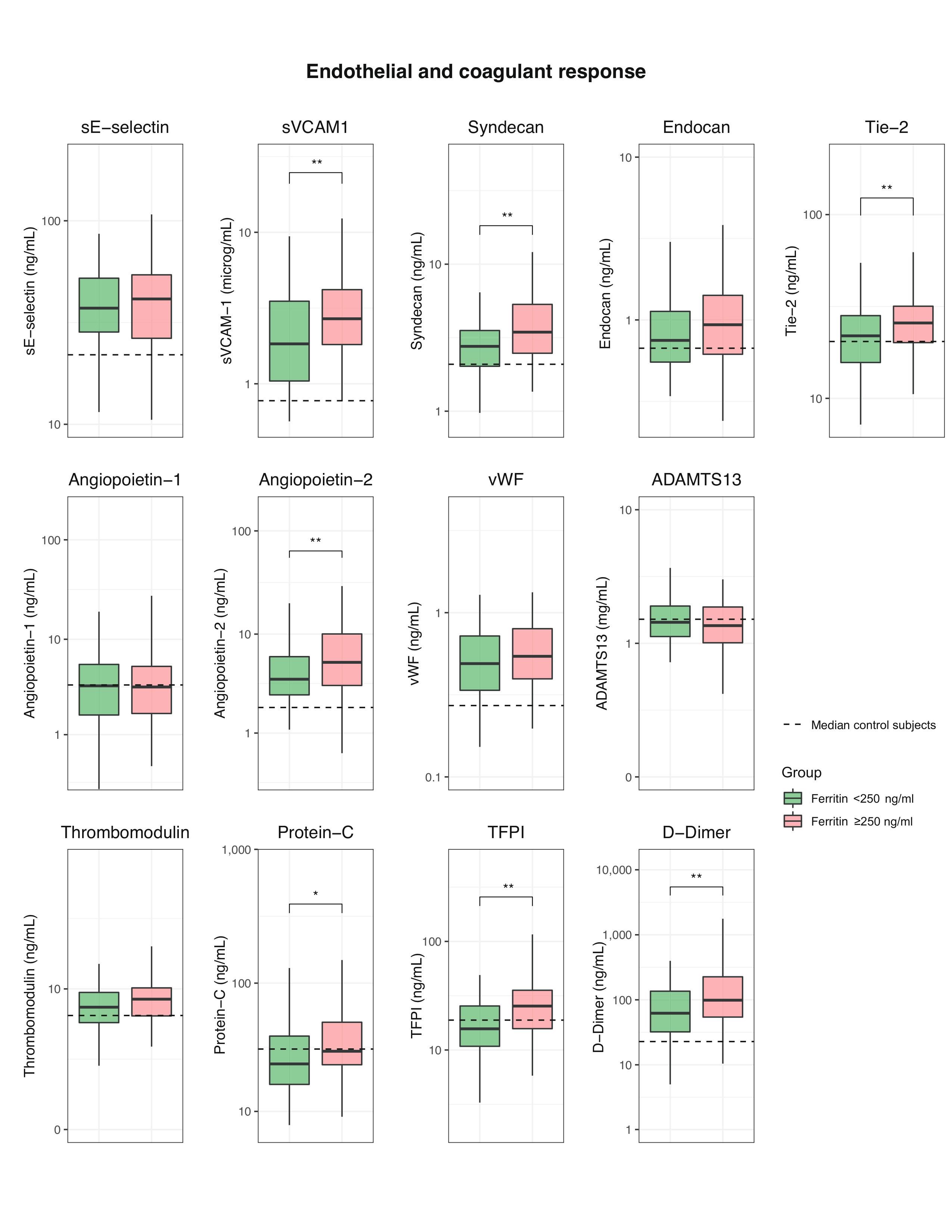

Supplement: jiac013_suppl_Supplementary_Figrue_S3 [file jiac013_suppl_supplementary_figrue_s3.jpeg]
