# Supplementary material for: Association of Hyperferritinemia With Distinct Host Response Aberrations in Patients With Community-Acquired Pneumonia
Source: J Infect Dis. 2022 Jan 31;225(11):2023–32. doi: 10.1093/infdis/jiac013 (PMC9312861; doi:10.1093/infdis/jiac013)
Supplement: jiac013_suppl_Supplementary_Data [file jiac013_suppl_supplementary_data.docx]

**SUPPLEMENTARY FIGURE LEGENDS**

**Supplementary Figure 1. Systemic inflammatory and neutrophil responses of patients with CAP stratified according to a plasma ferritin concentration of <250 ng/ml or ≥250 ng/ml.** Patients were stratified into those with plasma ferritin <250 ng/ml (n = 84) or ≥250 ng/ml (n = 90). Data are expressed as box-and-whisker diagrams with the horizontal line depicting the median, the top and bottom of the box representing the lower and upper quartiles, and whiskers extending to the farthest points that are not outliers (i.e., that are within 1.5 times the interquartile range of the lowest and the highest quartile, respectively). Dotted lines represent median values obtained in 50 age- and sex matched subjects. Asterisks indicate differences between groups (Benjamini-Hochberg corrected, **p* <0.05, ***p* <0.01). CRP = C-reactive protein; sTREM = soluble triggering receptor expressed on myeloid cells; sCD = soluble cluster of differentiation; MPO= myeloperoxidase; NGAL = neutrophil gelatinase-associated lipocalin.

**Supplementary Figure 2. Plasma cytokine levels in patients with CAP stratified according to a plasma ferritin concentration of <250 ng/ml or ≥250 ng/ml.** Patients were stratified into those with plasma ferritin <250 ng/ml (n = 84) or ≥250 ng/ml (n = 90). Data are expressed as box-and-whisker diagrams with the horizontal line depicting the median, the top and bottom of the box representing the lower and upper quartiles, and whiskers extending to the farthest points that are not outliers (i.e., that are within 1.5 times the interquartile range of the lowest and the highest quartile, respectively). Dotted lines represent median values obtained in 50 age- and sex matched controls. Asterisks indicate differences between groups (Benjamini-Hochberg corrected, **p* <0.05, ***p* <0.01). IL = interleukin; RA = receptor antagonist.

**Supplementary Figure 3. Endothelial and coagulant responses of patients with CAP stratified according to a plasma ferritin concentration of <250 ng/ml or ≥250 ng/ml.** Patients were stratified into those with plasma ferritin <250 ng/ml (n = 84) or ≥250 ng/ml (n = 90). Data are expressed as box-and-whisker diagrams with the horizontal line depicting the median, the top and bottom of the box representing the lower and upper quartiles, and whiskers extending to the farthest points that are not outliers (i.e., that are within 1.5 times the interquartile range of the lowest and the highest quartile, respectively. Dotted lines represent median values obtained in 50 age- and sex matched controls. Asterisks indicate differences between groups (Benjamini-Hochberg corrected, **p* <0.05, ***p* <0.01). s = soluble; sVCAM = soluble vascular cell adhesion molecule; vWF = von Willebrand factor; ADAMTS = a disintegrin and metalloproteinase with a thrombospondin; TFPI = tissue factor pathway inhibitor.
